# A Case of Spongiotic Osteoma Arising in the Supratubal Recess

**DOI:** 10.7759/cureus.82860

**Published:** 2025-04-23

**Authors:** Mikio Kuwabara, Yuko Matsumoto, Masaomi Motegi, Kazuaki Chikamatu

**Affiliations:** 1 Otolaryngology-Head and Neck Surgery, Gunma University Hospital, Maebashi, JPN; 2 Otolaryngology-Head and Neck Surgery, Gunma University Graduate School of Medicine, Maebashi, JPN

**Keywords:** middle ear osteoma, middle ear tumors, mixed hearing loss, spongiotic osteoma, tees

## Abstract

Osteoma of the middle ear is a rare condition in which tumors composed of mature bone grow. In the present study, we experienced a case of an osteoma causing the obstruction of the supratubal recess of the Eustachian tube, resulting in a depression in the pars flaccida of the tympanic membrane and fixation of the ossicles. The patient, a 37-year-old woman, had been aware of hearing loss for two years. Tympanic membrane findings showed a depression in the pars flaccida of the tympanic membrane, and audiometry showed mixed hearing impairment. A temporal bone computed tomography (CT) showed a spherical osteoma in the supratubal recess, with the ear ossicles adhering to the canopy with osteophytes. The patient underwent an endoscopic resection of the osteoma and tympanoplasty, and after two surgeries, her hearing improved. The osteoma in the supratubal recess was found to be a spongiotic osteoma. The osteophyte of the canopy was a compact osteoma. The slowly increasing spongiotic osteoma was thought to have exacerbated the ventilation of the epitympanum, leading to a depression of the pars flaccida of the tympanic membrane and the formation of compact osteoma due to inflammation.

## Introduction

Osteomas are benign tumors of mature bone. They are most commonly found in the head and neck region, with the skull, mandible, paranasal sinuses, and orbit being the predominant sites [1]. External auditory canal occurrence is relatively common in the temporal bone but rare in the middle ear [2]. In patients with progressive hearing loss, osteoma is a differential disease if there is a white lesion visible through the tympanic membrane, and the diagnosis can be confirmed by temporal bone computed tomography (CT) [3-5]. Osteoma in the supratubal recess is extremely rare [6,7], and we report on the findings in the ear and the course of treatment.

## Case presentation

The patient is a 37-year-old woman with a chief complaint of decreased hearing on the left side of her ear. She had been aware of left hearing loss since she was around 35. She was referred to our department with a suspected cholesteatoma with no history of recurrent otitis media.

**Figure 1:**
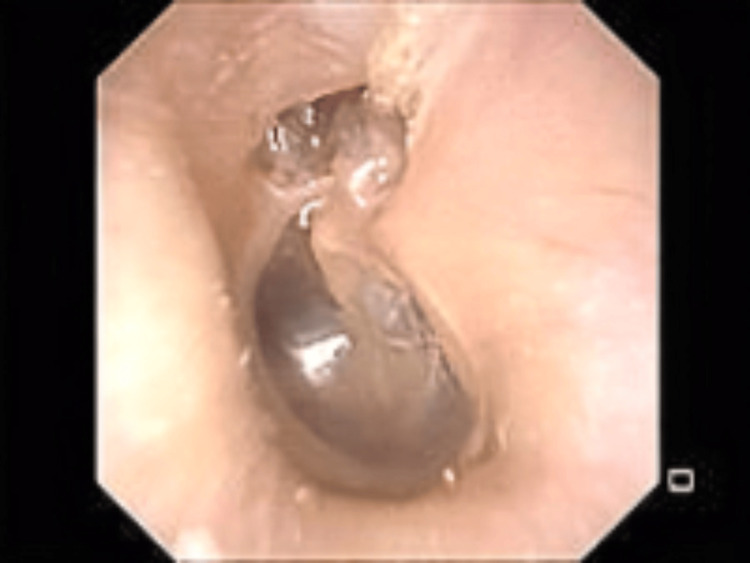
Left tympanic membrane findings on initial examination Depression in the pars flaccida of the left tympanic membrane.

**Figure 2:**
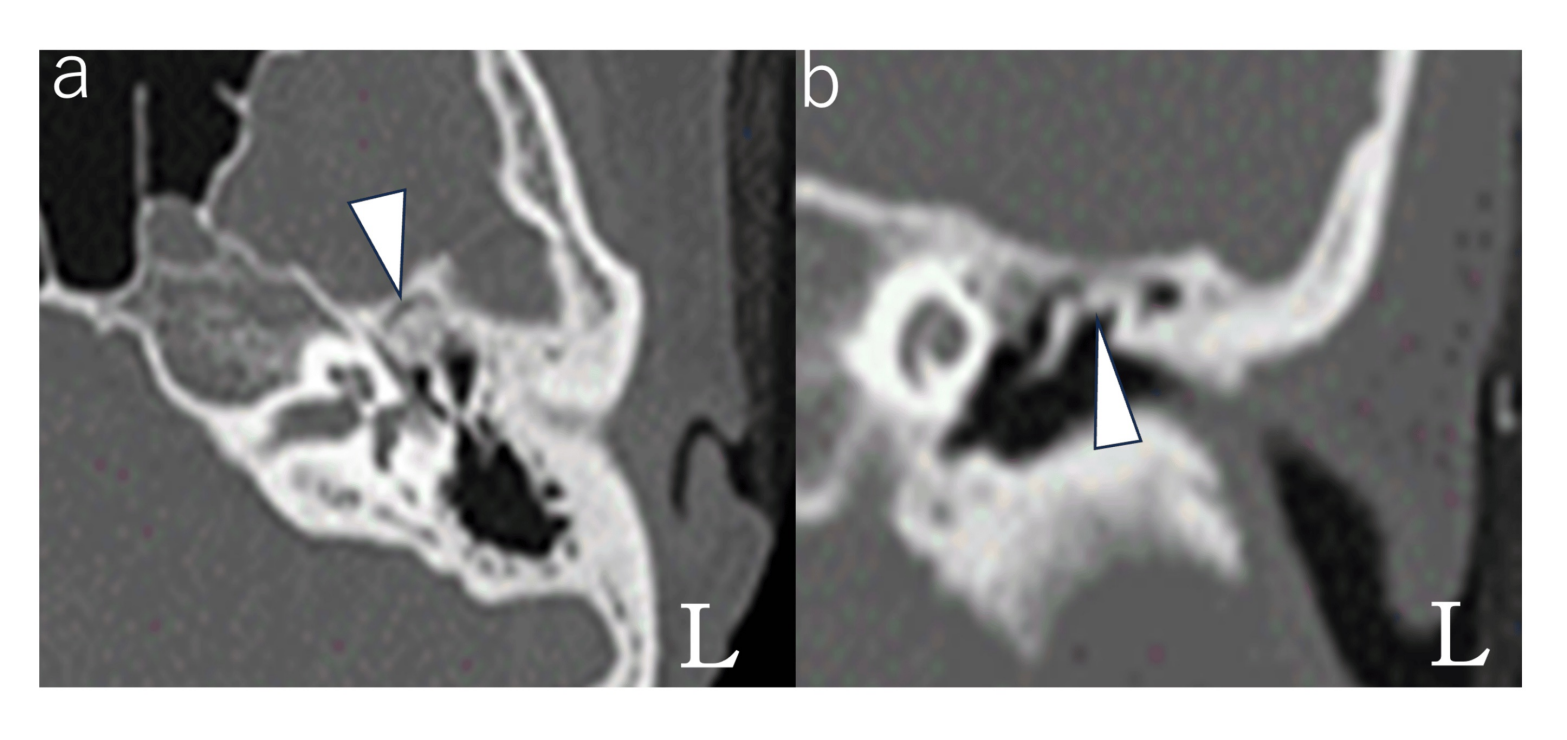
Computed tomography of the left temporal bone An axial computed tomographic image (a) showed a spherical bony lesion occupying a supratubal recess (arrow). A coronal computed tomographic image (b) showed a bony lesion extending from the tympanic canopy to the incus bone (arrow).

Depression was observed in the pars flaccida of the left tympanic membrane, but no abnormality was found in the pars tensa (Figure 1). There was no evidence of infection or keratosis in the depression. Audiometry revealed a left mixed hearing loss with a stiffness curve, which was similar to the audiogram of tympanosclerosis. Temporal bone CT showed a high-absorption spherical mass with an internal low-absorption zone in the left supratubal recess and a high-absorption beam extending from the tympanic cavity canopy to the incus bone (Figure 2).

The lesions were considered osteomas of the supratubal recess and the bony beams of the upper tympanic chamber. Hearing tests in this case are similar to those for otosclerosis and tympanosclerosis; these bony lesions restrict the range of movement of the ear ossicles, resulting in reduced hearing. The patient requested surgery to improve her hearing.

**Figure 3:**
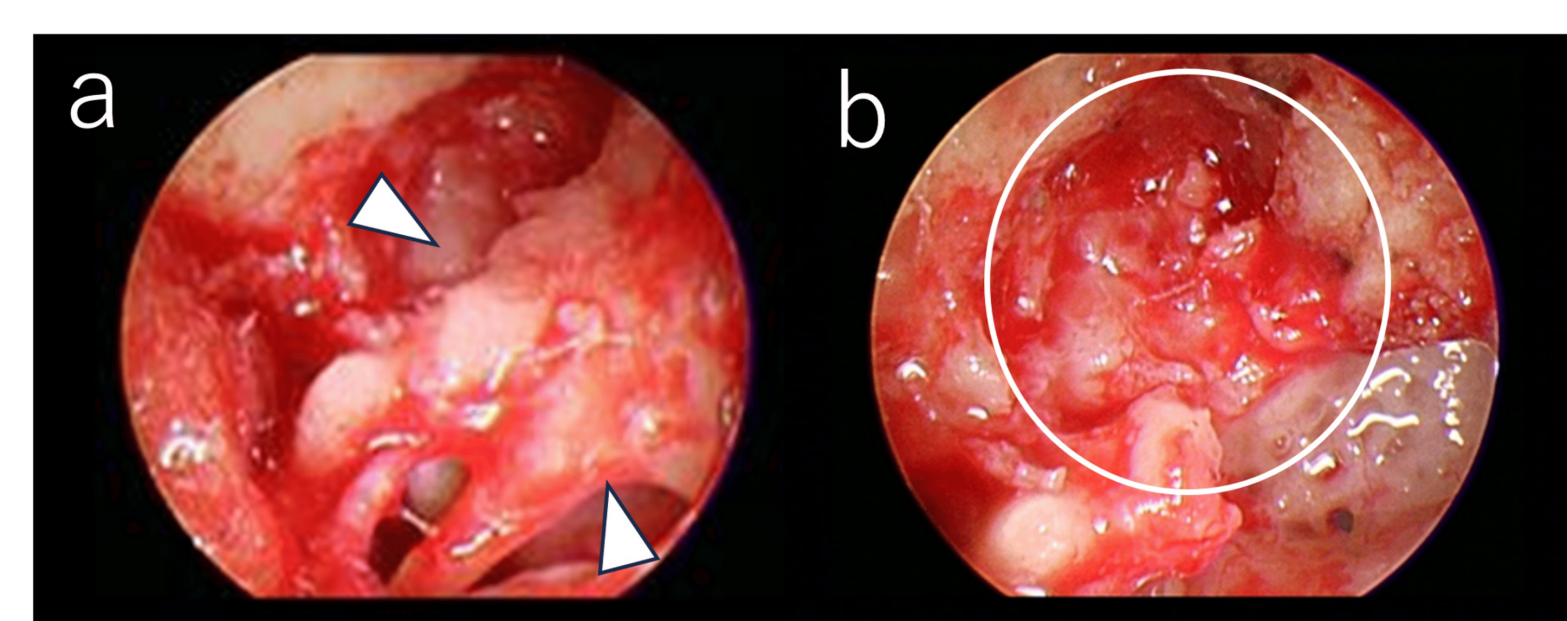
Intra-operative photograph (0° endoscope view) (a) The head of the malleus bone and the body of the incus bone are adhered to the tympanic canopy by the osteophytes (arrow). (b) Image after removing the head of the malleus bone and the body of the incus bone. The supratubal recess is obstructed by an osteoma (circle).

Endoscopically, the external auditory canal skin and tympanic membrane were elevated, and the capitulum angle was removed. The malleus and incus bones were adhered to the canopy by the osteophytes and were not mobile (Figure 3a). The bony beams were removed using a chisel, and the heads of the malleus and incus bones were removed. The supratubal depression was closed using a bone mass (Figure 3b). It was sherbet-like in consistency and was removed as much as possible with a sharp bone spoon and bar. The tumor in the supratubal recess was relatively hemorrhagic.

Mobility of the stapes was good. A columella made from the incus bone was placed between the handle of the malleus and the stapes head to reconstruct the sound transmission. The facial nerve was not intra-operatively exposed. No post-operative dizziness or facial nerve palsy was observed.

**Figure 4:**
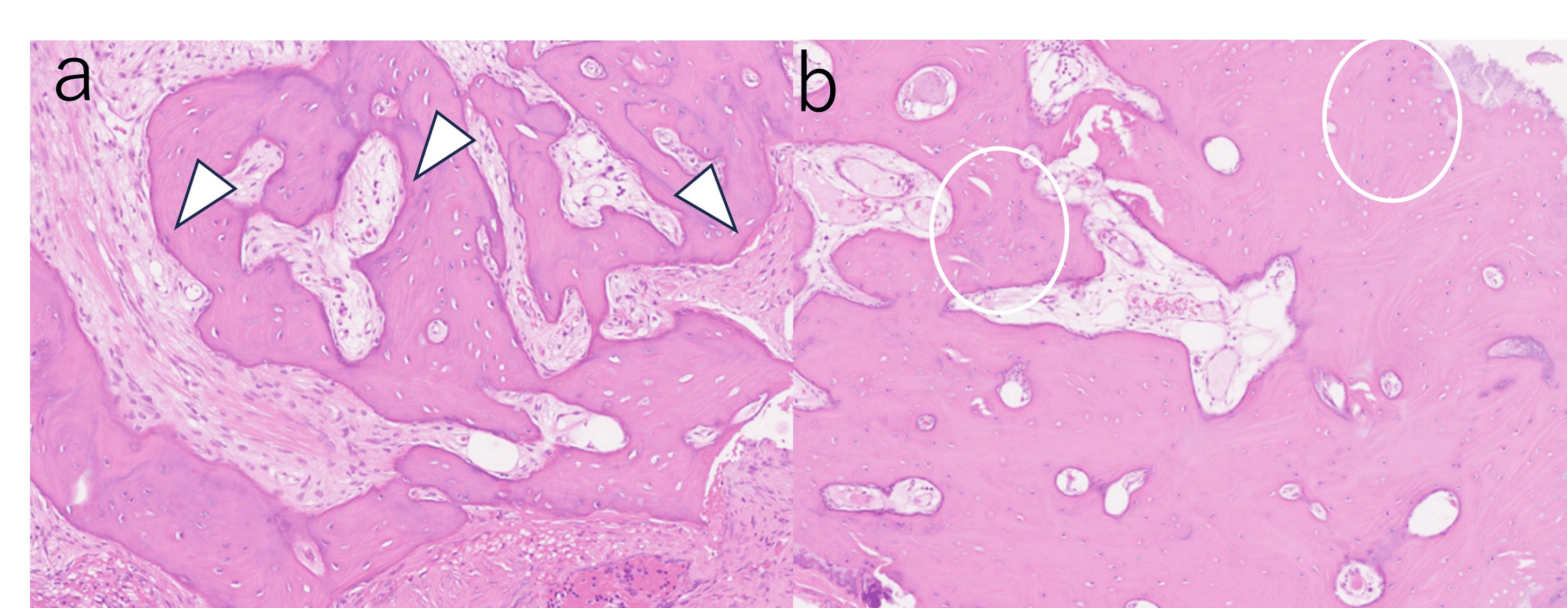
Histopathological findings Osteoma of the supratubal recess (a): laminar arcuate bone growth with fibrous connective tissue (arrow). Osteoma of the canopy (b): laminar bone proliferation with mild infiltration of inflammatory cells (circle) (stained with hematoxylin and eosin ×40).

On histopathological examination, the osteoma of the supratubal recess was diagnosed as spongiotic osteoma, as it showed a mixture of laminar arcuate bone and fibrous connective tissue (Figure 4a). In contrast, the osteoma of the canopy was thought to be a compact osteoma due to the lack of fibrous connective tissue. An infiltration of inflammatory cells was observed there (Figure 4b).

**Figure 5:**
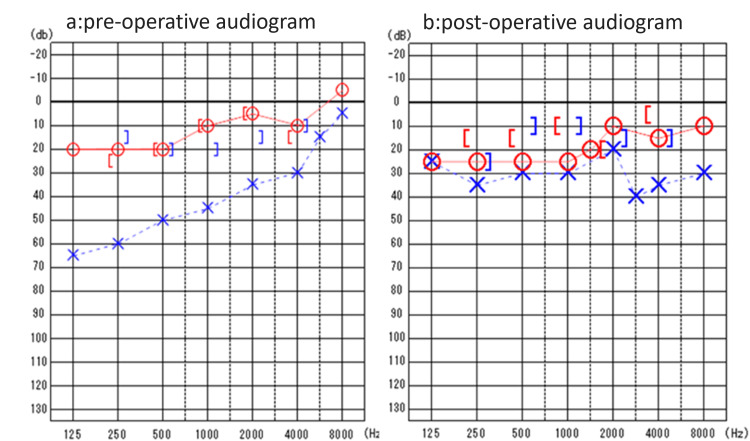
Pure tone audiograms Graphs showing (a) pre-operative pure tone audiometry result, 45 dB air-conduction threshold with 25 dB air-bone gap, and (b) post-operative pure tone audiometry result, 30 dB air-conduction threshold with a 15 dB air-bone gap. Bone conduction thresholds also showed an improvement from 20 dB to 10 dB (1000 Hz).

Unexpectedly, no improvement in hearing was achieved. Therefore, a second operation was performed one year later. After changing to a cartilage columella and adjusting its position, good post-operative hearing was achieved (Figure 5).

Currently, 36 months after surgery, no recurrence has been observed.

## Discussion

Osteoma of the middle ear was first reported by Thomas in 1964 [8], and 44 cases reported between 1964 and 2019 were summarized by Yoon et al. and Temirbekov and Celikyurt [6,7]. According to this report, middle ear osteoma was more common in women (61%), and the age of onset ranged from five to 57 years, with a mean age of onset of 28 years. The most common site of occurrence was the capillary angle of the tympanic chamber (17 patients, 38.6%), followed by the ossicles (eight patients, 18.1%), epitympanum (six patients, 13.6%), pyramidal ridge (four patients, 9.1%), hypotympanum (three patients, 6.8%), and lateral semicircular canal (three patients, 6.8%). Considering articles in English, there have been no reports of cases where the supratubal recess was obstructed by an osteoma, as in the present case.

Osteomas are histopathologically classified as spongiotic osteomas, consisting of cancellous bone with a marrow cavity and fibrous tissue; compact osteomas consisting of layered bone without a marrow cavity; or mixed forms of both [1]. Spongiotic osteomas are slow-growing, arborescent, and solitary osteomas. Compact osteomas have a broad base and grow relatively rapidly. The cause of osteomas remains unknown; however, it is thought to be congenital, inflammatory, or traumatic [1,9]. Spongiotic osteomas are often considered congenital owing to their structural complexity; however, it is difficult to identify the cause by histological examination alone, as inflammatory osteomas may also show features of spongiotic osteomas. Therefore, a comprehensive decision regarding the course and nature of the disease is necessary [10].

In the present case, the patient was aware of hearing loss for two years, and it is thought that the ear ossicles became adherent then. The timing of the development of spongiotic osteoma of the supratubal recess is unknown. However, it is likely to be congenital as there has been no history of repeated infection or trauma. Normally, air reaches the middle ear from the pharynx through the Eustachian tube and fills the epitympanum via the supratubal recess [11]. It is speculated that as a result of the gradual enlargement of the spongiotic osteoma, sufficient air was no longer able to enter the epitympanum, which promoted the collapse of the pars flaccida of the tympanic membrane and the formation of compact osteoma due to inflammation.

The initial symptom of most middle ear osteomas is hearing loss. In most cases, hearing can be improved by surgery [6,7]. As osteoma increases slowly, follow-up is the treatment of choice in asymptomatic cases, but there have been reports of facial nerve paralysis due to the compression of the middle ear osteoma [12]; therefore, surgical resection was considered significant to prevent paralysis. Cases of implantation of a bone conductive hearing aid have also been reported, which may be a future option in cases where resection is difficult [3]. In this case, surgery was performed to improve hearing; however, the initial operation was unsuccessful. During the second operation, the cartilaginous columella was placed in contact with the tympanic membrane, and hearing improved. The movement of the stapes was good, and the poor mobility of the handle of the malleus due to inflammation was considered to be one of the reasons why the hearing did not improve.

## Conclusions

Among the rarest middle ear osteomas, we encountered a rarer spongiotic osteoma of the supratubal recess. A depression was found in the pars flaccida of the tympanic membrane, and the ear ossicles were adhered to the canopy by the osteophytes. Osteoma removal and hearing improvement surgery was performed endoscopically under general anesthesia, and the mixed hearing loss improved after two operations. The case reaffirms the importance of detailed pre-operative CT reading and careful surgical planning.
